# Miscellaneous Notes

**Published:** 1880-03

**Authors:** 


### MISCELLANEOUS NOTES.

The humble red cabbage in the hands of the chemist, by maceration and pressure, produces an innocuous coloring matter called

Cauline, it forms the base for several colors when treated with re-agents. A fish called the *mahsir*, has been heard to utter a peculiar "cluck," and we suggest that as fish are now known to possess a voice, we may be able to get at the truth in fish stories. ——Ohio has 726.580 male inhabitants, over twenty-one years of age, and 1,044.479 school-youths, between the ages of six and twenty-one years.——This country produced nearly 20,000.000 barrels of crude petroleum last year, an increase of 5,000.000 bbls. over any previous year.——An Eastern publishing firm has begun the publication of what they call the Humboldt Library, consisting of papers on popular science from the best authors, the numbers cost but a few cents a piece, and should be had by all. ——The increase in value of last year's crops of cotton, sugar and tobacco, over those of the previous year, is placed $50,000.00.—— The anxiety of the presidents of some of our leading Universities and Colleges, to disclaim the teaching of evolutionary doctrines in their institutions is amusing.——The chestnut, when partially baked and ground into meal, is said to make a not unpalatable article of diet—good as saw-dust pudding anyhow.——Famine and Diptheria are decimating the population in some of the Russian districts.——A calculating machine invented by Leibnitz, in the 17th century, and lost shortly after, has recently been found and returned to the Hanover Public Library, where it originally belonged.——John Mears, F. R. S., an eminent English botanist, died in London, October 17th, 1879, at 91 years of age.-——The Esquimaux at Behring's Straight are reported as starving and dying in large numbers, the trouble is the scarcity of Walrus-meat, owing to the unwarranted destruction of these animals by American whalers.——"Creosoted piles driven at Portsmouth, England, forty-two years ago, are now as good above as below the water-line, and have out-lasted sixteen sets, cut from the same timber and driven in the same work, but which were not creosoted."——In January last, John Hoey, Esq., at Hollywood, N. J., exhibited a century plant in full bloom, at only twelve years of age.——Prof. B. F. Mudge, died at his home, Manhattan, Kansas, on 21st November last, his writings and re-searches in geology and paleontology placed him high amongst American scientists.——The highest railway is that a cross the Andes Mountains, at an elevation of 15,500 feet.——James Clerk Maxwell of the University of Cambridge, Dr. Karl Frederich Mohr of Bonn, and Wm. W. Saunders of London, have all died within the past few months. ——At Waldorf, near Bonn, Germany, extensive excavations have revealed the site of an old Roman town.——The clerk of the weather will be at his wits end to explain away this peculiar winter.

BROOKLYN, N. Y., Feb. 12th, 1880.

At a regular meeting of the Brooklyn Dental Society, held Feb. 9th, 1880, the following report of special committee, expressive of the sentiments of the society, in relation to the death of Dr. Samuel Stockton White, of Philadelphia, who died in Paris, Dec. 30th, 1879, was unanimously adopted.

"When the sad intelligence flashed across the sea from a foreign land, that death had removed from us, our honored friend and brother in the full maturity of his manhood and the height of his usefulness, our first thought was: Why should such a man, so identified with the interests of our profession, so active and useful in the cause of charity and religion, be taken away at the very summit of his power?

Dr. White was a man whom we had all learned to love and honor for his many manly virtues and his sterling integrity. In his death the dental profession has lost one who was always ready to aid any movement for the advancement of its best interests.

Although lately he has not been identified with the profession as a practitioner, his position as a manufacturer of the many appliances needful in our practice, had brought him into communication with the humblest as well as the highest in our ranks, and all can testify to his clear comprehension of what was necessary to our use, his willingness to aid those struggling in adversity, and his general interest in everything pertaining to the wants of our profession.

The dental profession throughout the world will morn his loss. Our several dental societies will miss his magnetic influence, and each individual dentist will feel that he had lost a friend and a brother.

His memory will always be to his family everything that is noble and lovable and we tender to them our heartfelt sympathy in this hour of their bereavement.

C. P. CRANDELL,
Rec. Sec., Brooklyn Dental Society.

